# PCR-DGGE assessment of the bacterial diversity of breast milk in women with lactational infectious mastitis

**DOI:** 10.1186/1471-2334-8-51

**Published:** 2008-04-18

**Authors:** Susana Delgado, Rebeca Arroyo, Rocío Martín, Juan M Rodríguez

**Affiliations:** 1 Dpt. Nutrición, Bromatología y Tecnología de los Alimentos, Universidad Complutense de Madrid, 28040 Madrid, Spain

## Abstract

**Background:**

Infectious mastitis is a common condition during lactation and in fact, represents one of the main causes leading to a precocious weaning. The number of studies dealing with lactational mastitis is low and, up to now, the etiological diagnosis is frequently made on the basis of unspecific clinical signs. The aim of this study was to investigate the microbial diversity of breast milk in 20 women with lactational mastitis employing culture-dependent and culture-independent (PCR-DGGE) approaches.

**Methods:**

Breast milk samples were cultured in different media to investigate the presence of bacteria and/or yeasts, and a total of 149 representative isolates were identified to the species level by 16S rRNA gene PCR sequencing. The microorganisms recovered were compared with those found by PCR-DGGE analysis. To identify the DGGE profiles two reference markers of different microbial species were constructed. Sequence analysis of unknown bands was also performed.

**Results:**

Staphylococci were the dominant bacterial group and *Staphylococcus epidermidis *was the dominant species. In a lower number of samples, other bacteria (mainly streptococci and a few gram-negative species) were also identified. Globally, PCR-DGGE results showed a good correlation with those obtained by culture-based methods. However, although DNA bands corresponding to different lactic acid bacteria were detected, such bacteria could not be isolated from the milk samples.

**Conclusion:**

Staphylococci seem to be the main etiological agents of human lactational mastitis. The combined use of culture and molecular techniques allowed a better characterization of the bacterial diversity in milk from women suffering from infectious mastitis. Our results suggest that this condition could be the result of a disbiotic process where some of the bacterial species usually present in human milk outgrow (staphylococci) while others disappear (lactobacilli or lactococci).

## Background

Infectious mastitis is a common condition during lactation and represents one of the main medical causes of precocious weaning [1,2] due to the associated symptoms, including fever, pain and/or tiredness. Since the WHO recommends breastfeeding during, at least, the first 6-months of life because of the health benefits that it provides to the mother-child pair [2], lactational mastitis could actually be considered as a public health issue.

Although the number of studies dealing with human lactational mastitis is low [1], *Staphylococcus aureus *and some streptococcal species have been traditionally considered as the main etiological agents [3]. In addition, the web pages of many associations that promote breastfeeding claim that yeasts are involved in infectious mastitis and sore nipples, but they do not provide any scientific evidence backing such affirmation. In fact, and despite the advertences highlighting the need to analyze and cultivate human milk [4], the etiological diagnosis of infectious mastitis cases is frequently made on the sole basis of unspecific clinical signs [5,6]. This situation contrasts with that in Veterinary Medicine, where infectious mastitis leads to important economical losses in dairy farms and, therefore, microbiological and somatic cell analyses are performed routinely.

In the last years, it has been shown that breast milk is an important source of bacteria to the infant gut where they play a key role in the initiation and development of the gut microbiota. Bacteria commonly isolated from this biological fluid in healthy women include staphylococci, streptococci, lactococci, lactobacilli and enterococci [7,8]. The presence of commensal and/or potentially probiotic bacteria in milk seems to be the result of the colonization of the mammary ducts and areola by maternal gut bacteria during pregnancy and lactation [9,10]. Such bacteria reach the mammary gland through the entero-mammary pathway.

In this study, we combined culture- and molecular-based methods to test the hypothesis that infectious mastitis may be the consequence of a disbiotic process leading to an overgrowth of certain bacterial species. To our knowledge, this is the first study applying molecular techniques to analyze the bacterial diversity in milk obtained from women suffering lactational mastitis.

## Methods

### Subjects and sampling

A total of 20 women aged 26–34 years with clinical symptoms of infectious mastitis participated in the study. They were diagnosed by the lactation consultants attending different primary health-care centers in Spain and met the following criteria: breast redness and pain, flu-like symptoms (including fever ≥ 38.5°C) and a leukocyte count higher than 6 log_10 _cfu mL^-1^. Women with mammary abscesses or any kind of parallel diseases were excluded from the study. All volunteers gave written informed consent to the protocol, which was approved by the Ethical Committee of Hospital Clinico of Madrid (Spain).

To collect the milk samples, nipple and mammary areola were cleaned with soap and sterile water, and then chlorhexidine was applied. The breast milk sample was collected in a sterile tube after manual expression using sterile-gloves. The first drops (approximately 250 μL) were discarded to avoid chlorhexidine contamination

### Isolation and enumeration of breast milk bacteria

Proper dilutions of the fresh breast milk samples were plated onto ready-to-use Baird Parker (BP; a selective medium for staphylococci isolation), Columbia Blood Agar (CNA; a medium particularly suitable for isolation of streptococci, staphylococci and related bacteria), and Sabouraud-Chloramphenicol (SDC; yeast isolation) agar plates supplied by BioMerieux (Marcy l'Etoile, France), and also on Kanamycin aesculin azide (KAA; Oxoid Ltd., Basingstoke, UK; isolation of enterococci), MRS (Oxoid; isolation of lactic acid bacteria) and Violet red bile glucose (VRBGA; Difco, Detroit, MI, USA; isolation of enterobacteria and other Gram-negative bacteria) plates. The plates were incubated at 37°C for 48 h with the exception of the SDC ones which, parallel, were incubated at 25°C for 5 d. The counts were performed by triplicate.

### Identification of breast milk bacteria

Between 4 and 10 different colonies were isolated from each sample. Globally, 149 isolates were identified to the species level by a combination of classical tests (morphology, Gram-staining, catalase, oxidase and coagulase assays) and 16S rRNA gene PCR sequencing using primers pbl16 (5'-AGAGTTTGATCCTGGCTCA-3') and mlb16 (5'-GGCTGCTGGCACGTAGTTAG-3') [11] and the BioredMix system (BioLine, London, UK). PCR conditions were as follows: 96°C for 4 min, 30 cycles at 96°C for 30 s, 50°C for 30 s, and 72°C for 45 s, and a final extension step at 72°C for 4 min. Amplicons were purified using the Nucleospin^® ^Extract II kit (Macherey-Nagel, Düren, Germany) and sequenced at the Genomics Unit of the Universidad Complutense de Madrid, Spain. The resulting sequences were used for searching sequences deposited in the GenBank database using the BLAST program and the identity of the isolates was determined on the basis of the highest scores (>98%).

### DNA extraction from milk samples and PCR-DGGE conditions

Total DNA was isolated from the milk samples using the QIamp DNA stool mini kit (QIAgen, Hilden, Germany) with the modifications described by Martin et al. [12]. Purified DNA was used as a template to amplify the V6 to V8 region of eubacterial 16S rDNA with primers U968-GC (5'-CGC CCG GGG CGC GCC CCG GGC GGG GCG GGG GCA CGG GGGG AAC GCG AAG AAC CTT AC-3') and L1401-r (5'-CGG TGT GTA CAA GAC CC-3') [13]. The underlined sequence in U968-GC corresponds to the GC-clamp. PCR and DGGE analysis were performed as described previously [12]. Optimal separation of the PCR products for the species detected in milk samples was achieved by a 30 to 55% urea-formamide denaturant gradient gel. Electrophoresis was performed in a Dcode System apparatus (Bio-Rad). The DNA bands were visualized by silver staining and developed as previously described Sanguinetti et al. [14].

### Identification of DGGE bands

**Figure 1:**
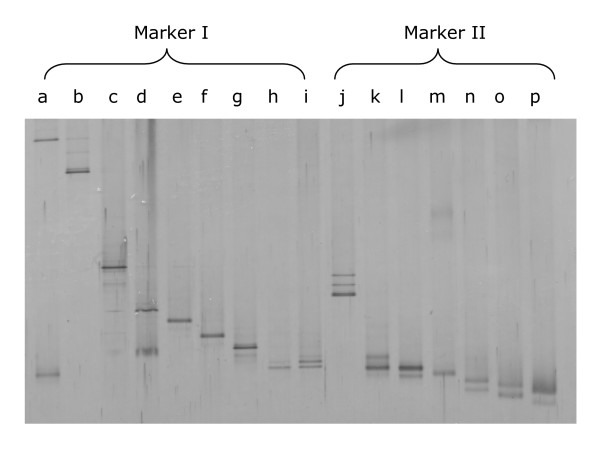
**DGGE profiles of the 16S rDNA amplicons used to construct the DGGE markers.** Lanes: a, *Staphylococcus epidermidis*; b, *Staphylococcus aureus*; c, *Klebsiella oxytoca*; d, *Pseudomonas fluorescens*; e, *Streptococcus mitis*; f, *Streptococcus salivarius*; g, *Streptococcus oralis*; h, *Streptococcus parasanguis*; i, *Rothia mucilaginosa*; j, *Lactobacillus salivarius*; k, *Enterococcus faecalis*; l, *Enterococcus faecium*; m, *Lactobacillus reuteri*; n, *Lactobacillus johnsonii*; o, *Lactobacillus gasseri*; p, *Lactobacillus fermentum*.

The bands observed in the DGGE were identified using two different approaches. The first consisted of the comparison of the DGGE profiles of the samples with those of two DGGE markers constructed previously (Figure 1). For their construction, chromosomal DNA of pure cultures of a variety of bacterial species was isolated using the DNA Tissue kit (QIAgen). Then, the chromosomal DNA was used for PCR amplification using primers U968-GC and L1401-r, as described above. The amplicons were purified and equal amounts were mixed to obtain the DGGE markers. Marker I comprised amplicons from (in migration order) *S. epidermidis*, *S. aureus, Klebsiella oxytoca*, *Pseudomonas fluorescens*, *Streptococcus mitis*, *Streptococcus salivarius*, *Streptococcus oralis*, *Rothia mucilaginosa *and *Streptococcus parasanguis *isolates. Marker II comprised amplicons from (in migration order) *Lactobacillus salivarius*, *Enterococcus faecalis*, *Enterococcus faecium*, *Lactobacillus reuteri*, *Lactobacillus johnsonii*, *Lactobacillus gasseri *and *Lactobacillus fermentum *isolates. Both markers were representative of some of the bacterial species frequently isolated from milk of healthy and/or mastitic women.

In addition, notorious DGGE bands that could not be identified by comparison with the DGGE markers were excised from the gels with sterile surgical blades and the DNA was extracted using the protocol of Sanguinetti et al. [14]. Extracted DNA was used as a template for PCR reamplification using the primers U968 (devoid of the GC-clamp) and L1401-r. The PCR products were purified using the Nucleospin^® ^Extract II kit and sequenced. Sequences were compared to those present in the databases using BLAST algorithm and their identity was determined on the basis of the highest scores.

## Results

### Isolation of bacteria from the milk samples

Globally, CNA and BP counts in the milk samples oscillated between 3.50 to 5.87 log_10 _cfu mL^-1 ^and, with the exception of sample M13, no statistically significant differences between CNA and BP counts were found (data not shown). This suggested that most of the CNA isolates actually belong to the genus *Staphylococcus*. In fact, identification of 4-10 different colonies per sample confirmed that staphylococci were the predominant bacteria in these samples since they were present in 18 samples (90%) and 104 (70%) of the identified isolates belonged to this genus (Table 1). At the species level, *S. epidermidis *was predominant since it was detected in 17 (85%) samples and 72 (48%) of the isolates belonged to this species (Table 1). Although not to the extent of *S. epidermidis*, *S. aureus *was the second predominant species (5 samples; 14 isolates), and some *Staphylococcus hominis*, *Staphylococcus pasteuri*, *Staphylococcus warneri*, *Staphylococcus lugdunensis *and *Staphylococcus haemolyticus *isolates were detected occasionally (Table 1).

**Table 1:** Bacterial species isolated from the milk samples (the data represent the number of isolates that were identified to a particular species within each sample)

**Species**	**Sample**																				
	
	M 1	M 2	M 3	M 4	M 5	M 6	M 7	M 8	M 9	M 10	M 11	M 12	M 13	M 14	M 15	M 16	M 17	M 18	M 19	M 20	Total
*Staphylococcus epidermidis*	4*	5*			4*	3*	6*	6*	1*	4*	4*	5*		4*	3*	3*	5*	4*	5*	6*	72

*Staphylococcus aureus*	4				1*		2*				3*			4*							14

*Staphylococcus pasteuri*					1				2								2		2		7

*Staphylococcus warneri*													2			2		1			5

*Staphylococcus hominis*						1			2											1	4

*Staphylococcus lugdunensis*													1								1

*Staphylococcus haemolyticus*													1								1

*Gemella haemolysans*								1*													1

*Rothia mucilaginosa*						2*				1*		3*						3*			9

*Rothia dentocariosa*																				1	1

*Kocuria kristinae*		1							2							1	2				6

*Streptococcus mitis*								1					1*						1*		3

*Streptococcus salivarius*													1*								1

*Streptococcus sanguinis*																	1				1

*Streptococcus parasanguis*								1*													1

*Streptococcus peroris*													1								1

*Enterococcus faecalis*												2*		1*	2*						5

*Corynebacterium pseudogenitalium*													1								1

*Klebsiella oxytoca*		2*	3*																		5

*Pseudomonas fluorescens*				5*																	5

*Enterobacter hormaechei*		1																			1

*Acinetobacter johnsonii*							1*														1

*Escherichia coli*				1																	1

*Pantoea agglomerans*			1																		1

*Delftia tsuruhatensis*					1																1

Number of isolates identified	8	9	4	6	7	6	9	9	7	5	7	10	8	9	5	6	10	8	8	8	149

**Figure 2:**
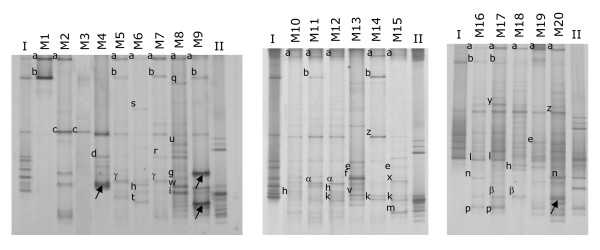
**DGGE analysis of the bacterial diversity in the milk samples.** Lanes: I and II, DGGE identification markers. DGGE marker I comprises the species listed from (a) to (i) in the Figure 1 legend while DGGE marker II contains the species listed from (j) to (p). M1 to M20, DGGE profiles of samples M1 to M20. Bands identified by gel excision and PCR sequencing are indicated by letters from (q) to (z) and from (α) to (γ), and correspond to the following bacteria: q, *Gemella haemolysans *[EMBL: AM774574]; r, *Acinetobacter sp. *[EMBL: AM774586]; s, *Staphylococcus capitis *[EMBL: AM774573]; t, *Arthrobacter *sp. [EMBL: AM774575]; u, *Streptococcus suis *[EMBL: AM774576]; v, *Streptococcus cristatus *[EMBL: AM774577]; w, *Streptococcus pneumoniae *[EMBL: AM774579]; x, *Streptococcus anginosus *[EMBL: AM774580]; y,*Lactobacillus animalis *[EMBL: AM774581]; z, *Lactococcus lactis *[EMBL: AM774582]; α, *Lactococcus garvieae *[EMBL: AM774583]; β, *Propionibacterium acnes *[EMBL: AM774584]; γ, *Neisseria weaveri *[EMBL: AM774585]. The arrows indicate the bands that matched with sequences identified as" uncultured bacterium DGGE band 16S ribosomal RNA gene" in the database.

*: Species that were also detected in each milk sample by PCR-DGGE (see Figure 2 for details).

Some of the CNA isolates belonged to the genera *Gemella*, *Rothia*, *Kocuria*, *Streptococcus *or *Corynebacterium *although they seemed to have a secondary role in respect to staphylococci, at least in quantitative terms (Table 1). The M13 sample displayed the widest spectrum of Gram-positive bacteria, including up to three different streptococcal species. In fact, this sample was the only one in which the CNA counts (4.65 log_10 _cfu mL^-1^) was significantly higher than the BP counts (3.06 log_10 _cfu mL^-1^).

The samples provided by women M3 and M4 were the only ones in which bacterial growth was not observed on CNA or BP plates; in these samples, colonies could only be isolated from VRBGA plates, where the counts were 3.20 and 4.79 log_10 _cfu mL^-1^, respectively. All VRBGA isolates were identified as Gram-negative bacteria, and the predominant species were *K. oxytoca *and *P. fluorescens *in samples M3 and M4, respectively (Table 1). VRBGA growth was also observed in samples M2, M5 and M7 although the counts were lower (between 1.70 and 2.40 log_10 _cfu mL^-1^). Gram-negative bacteria were isolated from these samples but staphylococci seemed to be predominant. Enterococci could only be isolated in KAA plates from samples M12 (2.08 log_10 _cfu mL^-1^), M14 (2.18 log_10 _cfu mL^-1^) and M15 (2.23 log_10 _cfu mL^-1^) while neither yeasts (SDC plates) nor lactobacilli or lactococci (MRS plates) could be detected in any sample. The partial 16S rRNA gene sequences obtained from the different isolates were deposited in the EMBL nucleotide sequence database under accession numbers [EMBL: AM779062] to [EMBL: AM779086].

### PCR-DGGE (denaturing gradient gel electrophoresis) analysis of breast milk from mastitis

The DGGE profiles obtained from the samples comprised between 2 and 9 predominant bands although some faint bands were also observed (Figure 2). The DGGE profiles seemed to be host-specific but the band corresponding to *S. epidermidis *was the most widely distributed since it was detected in 17 out of the 20 breast milk samples (Figure 2). The band corresponding to *S. aureus *was present in 8 samples (including 3 in which this species had not been detected by culture-based methods) and it was always accompanying the *S. epidermidis *band. The intensity of the *S. aureus *band was higher in the M1 and M14 profiles (Figure 2), which were the samples that provided the highest number of *S. aureus *isolates by using culture media (Table 1). In a few number of samples, DGGE-specific bands corresponding to other bacterial species, such as *S. mitis*, *S. oralis*, *S. salivarius*, *S. parasanguis*, *R. mucilaginosa*, *E. faecium*, *E. faecalis, L. reuteri, L. johnsonii*, *L. fementum, K. oxytoca *or *P. fluorescens*, were also present (Figure 2).

In addition, those dominant bands that were observed in the profiles but that did not match with any of the bands present in the DGGE markers were excised from the gels and used as templates for sequencing analysis. Following this procedure, DGGE bands corresponding up to 13 additional species were putatively identified (Figure 2). The 16S rRNA gene sequences were deposited in the EMBL nucleotide sequence database under accession numbers [EMBL: AM774573 to EMBL: AM774577; EMBL: AM774579 to EMBL: AM774586]. Additionally, the DNA from a few bands only matched with database sequences deposited as "uncultured bacterium DGGE band 16S ribosomal RNA gene" (Figure 2). Finally, we were not able to identify some minor bands because they could not be excised from the gels (too low intensity) or no amplification product was obtained from the excised fragment.

## Discussion

The role of specific agents in the etiology of human infectious mastitis has not been elucidated yet. Foxman et al. [1] published one of the most extensive reviews ever performed on lactational infectious mastitis and, although almost all the potential predisposing factors were carefully reviewed, no information was included regarding the etiological agents involved in each case. The authors justified such important omission on the basis of the few, and often antiquate, studies dealing with the subject and they stated that scientists should focus their attention on this problem because of its usually underrated medical and social impact.

In this study, 149 isolates obtained in different culture media from milk samples provided by 20 women were identified at species level by a combination of classical morphological and biochemical tests and by 16S rDNA PCR sequencing. Qualitatively (number of positive samples) and quantitatively (number of isolates in each sample), staphylococci were the main bacterial group isolated from the milk samples and in fact, 70% of the isolates belonged to this genus. Staphylococci are among the predominant bacteria in milk of healthy women but their concentration usually ranges from 1.5 to 3.0 log_10 _cfu mL^-1 ^(unpublished data). In contrast, most of the mastitis samples (75%) analyzed in the present study showed a staphylococcal count notably higher (≥ 4.0 log_10 _cfu mL^-1^). Most of the staphylococcal isolates were identified as *S. epidermidis*, a species considered as a normal inhabitant of the skin and mucosal surfaces in healthy hosts; in fact, its ubiquitous prevalence as a commensal species makes it difficult for a clinician to decide if an isolate represents the etiological agent or a culture contamination in a milk sample. Therefore, isolation ofthis bacterial species is generally regarded as non-related to a mastitis case, even when it is the only or predominant species present in a sample. In this context, the results of this work suggest that this bacterial species could be a widely underrated cause of lactational mastitis. Recent studies have shown the increasing importance of coagulase-negative staphylococci in bovine mastitis and have revealed that its incidence could be even higher than that of *S. aureus *[15,18]. Interestingly, we could isolate *S. epidermidis *from 17 of the 20 milk samples (85%) while *S. aureus *was only isolated from 5 samples (25%). Previously, it was suggested that *S. epidermidis *may be an etiologic agent of mastitis in nursing women, since the inoculation of strains isolated from human mastitis into the mammary glands of lactating mice led to clinical and histological signs of mastitis [19]. The complete genome analysis of *S. epidermidis *strains of human origin have revealed its propensity to cause chronic and recurrent infections [20], which are typical in most cases of lactational mastitis. Probably, *S. epidermidis *requires a predisposed host in order to change from a commensal inhabitant of the human body to an infectious agent [21,22]. This could explain why only a 3–30% of lactating women suffer from such infection whilst it is the predominant bacterial species found in breast milk of healthy women [7,12].

Streptococci are considered the second bacterial group involved in infectious mastitis [2]. However, in this study they were isolated only in 4 samples and always accompanied by outnumbering staphylococci. A total of 7 streptococcal isolates corresponding to 5 different streptococcal species could be isolated but none belonged to group B streptococci. These results suggest a secondary role of this bacterial group in the aetiology of infectious mastitis. Less frequently, other bacterial groups, such as enterobacteria, corynebacteria or mycobacteria, may also be involved [2]. In the present work, enterobacteria and other Gram-negative bacteria could be isolated from 5 samples and, in two of them (M3 and M4), they were the only bacteria that grew in culture media. With respect to yeasts, they could not be isolated from any of the samples. This observation coincides with the conclusions of a review of all the mastitis cases attributed to *Candida*, since it revealed that no microbiological analyses were ever performed [5]. The fact that *Candida*-associated mastitis has never been described in any other mammal, including cows, sheep or goats, where thousands of microbiological studies are performed every year, it is supportive as well.

PCR-DGGE analyses showed a good correlation with culture-based results concerning the following species: *S. epidermidis*, *S. aureus*, *Gemella haemolysans*, *R. mucilaginosa*, *S. mitis*, *S. salivarius*, *S. parasanguis*, *E. faecalis*, *K. oxytoca*, *P. fluorescens*, and *Acinetobacter johnsonii *(Table 1). On the other hand, DGGE bands corresponding to the rest of the staphylococcal species could not be identified despite the fact that they were isolated from culture media. However, there were some bands in the DGGE gels that did not correspond to any band in the markers, that were too faint to be excised from the gels or that did not produce any amplification product after the excision and purification (Figure 2). These bands may correspond to the cultured species for which no specific DGGE band could be assigned.

In addition, some of the bands present in the DGGE gels corresponded to bacterial species that could not be isolated. Among them, there were some lactic acid bacteria species (*Lactococcus lactis*, *Lactobacillus animalis*, *L. fermentum*, *L. johnsonii *and *L. reuteri*). Previously, such bacteria were identified in milk from healthy women both by culture- [7,8,10,23] and molecular-based methods [12] and have shown to display a high probiotic potential [23,24], including protection against mastitis-producing staphylococci [25]. The presence of lactic acid bacteria DNA could indicate that such bacteria were initially in milk but they were not longer viable after the overgrowth of the mastitis-causing agents. This suggests that infectious mastitis could be a disbiotic process similar to those occurring in the intestinal or vaginal ecosystems.

## Conclusion

This work confirms that staphylococci are among the main etiological agents of human lactational mastitis. In addition, our results suggest that this condition could be the result of a disbiotic process that alters the bacterial composition of human milk and leads to an outgrowth of certain bacterial groups, such as staphylococci. Although more studies are required, the results obtained in this work indicate that a combination of classical culture techniques and molecular microbiology procedures can be a good approach for a better understanding of the etiology of these infectious processes.

## Competing interests

The author(s) declares that they have no competing interests.

## Authors' contributions

SD carried out the milk analyses, the identification of the isolates and the PCR-DGGE assays and drafted the manuscript. RA assisted in the preparation of material and participated in the identification of the isolates and in the milk counts. RM set up the PCR-DGGE methodology. JMR conceived and coordinated the study, participated in its design and revised the manuscript. All authors have read and approved the final manuscript.

## Pre-publication history

The pre-publication history for this paper can be accessed here:


